# Sea-level rise projections tailored for spatial adaptation planning in the U.S.

**DOI:** 10.1038/s41597-026-06669-7

**Published:** 2026-07-15

**Authors:** Matthew A. Konfirst, Chris Feinman, Philip E. Morefield

**Affiliations:** 1 U.S. Environmental Protection Agency, Region 3, Philadelphia, USA; 2 Oak Ridge Institute for Science and Education, Oak Ridge, USA; 3 Department of Geography and Geoinformation Science, George Mason University, Fairfax, USA

**Keywords:** Projection and prediction, Environmental impact

## Abstract

We present a new geospatial dataset that describes both the extent and depth of inundation in the United States and outlying territories under global scenarios of sea-level rise. This new dataset incorporates projections from the 2022 National Oceanic and Atmospheric Administration (NOAA) technical report: Global and Regional Sea Level Rise Scenarios for the United States and integrates elements from the US Army Corps of Engineers (USACE) Sea-Level Rise Calculator. It provides users with intuitive access to relevant GIS data layers, enabling detailed analysis and visualization of potential scenario-based changes in mean sea level due to sea-level rise. This paper outlines the methodology, processing steps, and data validation involved in the dataset development, emphasizing the steps taken to further tailor information from the NOAA technical report to enhance the utility for spatial adaptation planning. These new geospatial datasets can provide critical insight for policymakers, researchers, planners, and others concerned with adapting to sea-level rise in coastal areas.

## Background & Summary

Sea-level rise poses a significant threat to coastal regions worldwide, but its impacts vary significantly across timescales, locations, and scenarios^1^. Within the United States alone, sea-level rise variability along the coastline presents a range of exposure to changes in local mean sea level^2^, all of which may necessitate an equally wide array of solutions to adapt to the effects of rising tides^3^. While sea-level rise is a well-documented phenomenon^4^, data regarding exposure to sea-level rise is not always available to stakeholders in coastal communities in a format that supports their specific needs^5^. There are numerous publicly available tools and data sources describing future sea-level rise, such as NOAA’s Sea-Level Rise Viewer (SLRV)^6^, NASA’s Sea-Level Rise Projection Tool^7^ and the US Army Corps of Engineer’s Sea Level Analysis Tool (SLAT)^8^. However, the presentation of sea-level rise data in these existing resources does not always address the needs of users engaged in professional adaptation work at local and regional scales. Furthermore, data and tools offered by non-governmental organizations may have limited access to data or include paywalls, which limit the ability of the user to assess exposure alongside other datasets or prohibit their use by smaller communities or organizations without the budget to access the full functionality of the tool. Novel tools and workflows for analyzing and visualizing potential impacts of sea-level rise are thus necessary to help users contextualize how changes to sea level will impact an area with respect to project-relevant timelines.

The work described here addresses this gap by using sea-level rise projections described in the 2022 NOAA technical report: Global and Regional Sea Level Rise Scenarios for the United States^2^ to produce a new set of high-resolution geospatial sea-level rise visualizations. The 2022 dataset was produced with a 1-degree resolution and at select tide gauge stations along all U.S. coastlines. The projections incorporate estimates of vertical land motion to produce a more thorough accounting of the factors impacting sea-level change at a specific location. The dataset is organized into five scenarios: 0.3 m (Low), 0.5 m (Intermediate-Low), 1.0 m (Intermediate), 1.5 m (Intermediate-High) and 2.0 m (High), with each scenario defined by the median change in Global Mean Sea Level (GMSL) in 2100 relative to the year 2000. Geospatial projections are provided for each scenario at the 17^th^, 50^th^ and 83^rd^ percentiles. Additionally, each scenario is assigned a probability of exceeding GMSL in 2100 based on global mean surface air temperature and cross referenced to standardized emissions scenarios known as Shared Socioeconomic Pathways. More information on the scenarios is available in the NOAA technical report cited above.

**Fig. 1:**
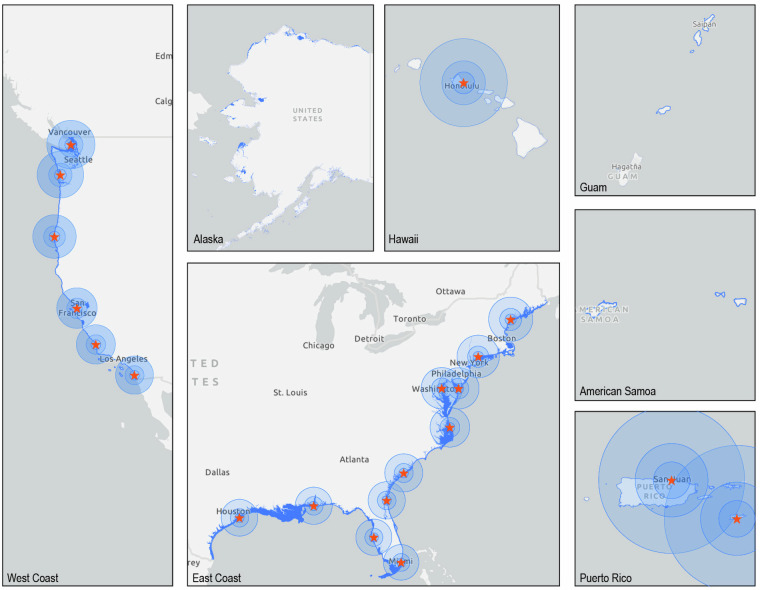
Sea-level rise data is available for all US coastlines, including CONUS, Alaska, Hawaii and each of the inhabited territories (American Samoa, Guam, Northern Mariana Islands, Puerto Rico, and the US Virgin Islands). Stars indicate locations referenced in the table in Table 1 and concentric circles represent the 25-, 50- and 100-mile (40.2, 80.5, and 160.9 km) data-validation buffers referenced in the text and Fig. 7. The shaded blue area represents inundation under the High scenario in the year 2100 and was chosen for illustrative purposes in this figure.

**Fig. 2:**
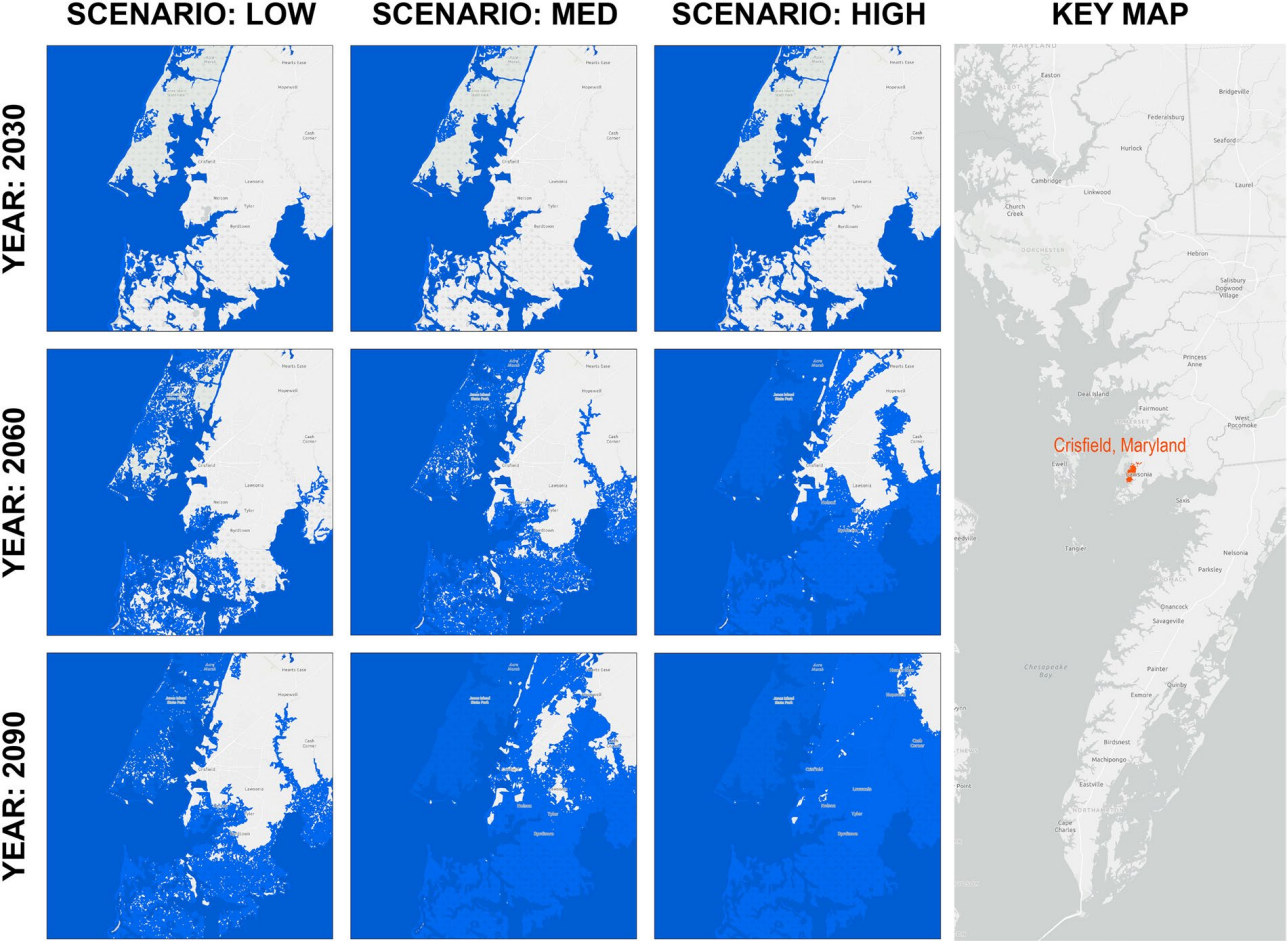
Data layers allow the user to visualize mean sea level under a range of sea-level rise scenarios and time horizons (with a total of 70 combinations). Depicted is Crisfield, MD along the eastern shore of Chesapeake Bay in 2030, 2060 and 2090. Low, med, and high correspond to the Low, Intermediate and High scenarios, described above.

The data products described in this manuscript include standardized GIS layers which represent the projected areal extent (polygons) and increased water depth (rasters) attributed to sea-level rise for all US coastlines, including US territories (Fig. 1). Consistent with the data available from the 2022 NOAA technical report, outputs were produced at decadal intervals from 2020 through 2150 using the median sea-level projections for each scenario, for a total of 70 scenario/decade combinations (Fig. 2). Raster data is calculated at 30-meter resolution, where each pixel represents an area that would likely be inundated under the specified GMSL scenario/decade horizon combination, and raster values indicate the average water depth for each pixel. This data has been processed according to the procedures described below and has been made available for download at https://zenodo.org/records/15265448^9^.

The advantage of presenting the data in this way is that users can intuitively select a sea-level rise scenario and time horizon to access a visualization that is most directly relevant to their decision-making needs. By design, interpreting the data is straightforward for viewers who are not versed in the scientific literature surrounding sea-level rise, largely because the source data used to create them has already integrated the variety of factors which contribute to this process. These factors include local variation, including topography, shifts in oceanographic factors such as circulation patterns, changes in the Earth’s gravitational field due to melting of land-based ice, vertical land movement (subsidence or uplift) due to glacial isostatic adjustment, sediment compaction, groundwater and fossil fuel withdrawals and other non-climatic factors, all of which have been built into the original NOAA projections. This makes it possible for users of these data products to observe regional variation in exposure without sacrificing the ability to analyze hyper-local conditions. Presenting tide and storm surge data alongside the GIS data will enable users to further tailor their analysis to their level of risk tolerance for potential projects and timelines.

Our workflow for generating these new geospatial layers is similar to that of other products in the sea-level rise analysis ecosystem, but it incorporates additional information such that the outputs are more useful for a specific audience: adaptation planners and practitioners. For example, the NOAA SLRV raster data also interpolates points to create continuous surfaces that represent the most precise representations of localized coastal dynamics possible. However, the NOAA dataset uses this technique only for the visualization of tidal phenomena, whereas sea-level rise itself is presented in 1-foot (0.30 m) increments above this surface, up to 10 vertical feet (3.05 m) of sea-level rise over mean higher high water (MHHW), independent of scenario and time. Users must then reference a data table based on tide gauge locations to understand differences between scenarios and connect the visualizations to time horizons, which may require a high baseline knowledge of sea-level rise dynamics to use the tool accurately. Additionally, NOAA sea-level rise data is often incorporated into other geospatial tools, such as EPA’s Cleanups in My Community^10^ and EnviroAtlas^11^ where it may be presented without valuable context needed by adaptation planners. For example, users may unknowingly select a highly improbable degree of sea-level rise, creating a potentially skewed representation of worst-case conditions for any given area over a project planning horizon. Given these constraints, it becomes difficult to use the data to answer questions involving magnitude, timing, and context for any given visualization.

**Fig. 3:**
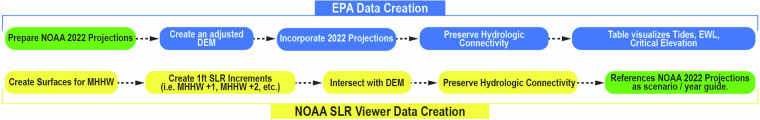
A comparison of the workflows used to develop the data described in this manuscript (top row, green/blue rectangles) and the data within the NOAA Sea-Level Rise viewer (bottom row, yellow/green rectangles). Note that the workflow described in this paper starts with the sea-level rise projections in the NOAA 2022 report (green) and focuses on the scenarios and time horizons. The NOAA Sea-Level Rise Viewer interprets the NOAA 2022 report data at the end of the process through the lens of map layers that depict Mean Higher High Water + x ft.

The datasets presented in this paper are organized by scenario and decade, meaning that data users cannot select arbitrary amounts of sea-level rise without reference to a particular pre-defined set of conditions (Fig. 3). Local factors influencing sea-level rise exposure (described above) are important to include when developing sea-level rise projections, but it is not necessary for decision makers to explicitly understand the scientific causes to make informed responses since these factors are included in the original projections. As an example, a small municipal planning department may want to perform a screening level analysis to select a location for a green infrastructure installation in their coastal township. The planners likely do not have working knowledge of sea-level-rise dynamics, but they do understand their own level of risk tolerance and the projected time horizon of their project investment. Since this dataset allows users to screen by scenario and by time, the planners can quickly create visualizations that allow them to analyze the most pertinent conditions without needing to consult a reference guide or becoming immersed in sea-level rise science.

To be sure, both methods of representing and analyzing this data are useful for a variety of purposes or audiences, and each tool has its strengths and weaknesses. Datasets found in NOAA’s Sea-Level Rise Viewer, NASA’s Sea-Level Rise Projection Tool, and the US Army Corps of Engineer’s Sea Level Analysis Tool, as well as those offered by non-governmental organizations, address the needs of diverse user groups, including academics, engineers and other advanced, technically proficient consumers of scientific information. In contrast, our data products are designed to be most useful to groups like state, federal, or local planners, community organizations, local landowners and developers, watershed organizations, environmental nonprofits, and other professionals whose work involves coastal adaptation planning, all of whom may not be as well versed in the scientific details of sea-level rise science but may nonetheless be interested in conducting a screening level assessment of their exposure.

## Methods

This paper focuses on the production of rasters (SLR depth) and shapefiles (SLR extent) organized by year and GMSL scenario, where each data file represents coastal inundation due to sea-level rise under a specified scenario and for a specific year (assuming there are no coastal protection measures not embedded in the underlying data). A series of modeling processes was developed to derive these products from the source data. The initial steps involve (1) defining an area for analysis, (2) preparing a Digital Elevation Model^11,12^ (DEM), and (3) using Triangulated Irregular Networks (TINs) to create raster files from sea-level rise point projection data^13^. The input layers were then used to (4) create gridded sea-level rise projections which were subsequently adjusted to (5) preserve hydrologic connectivity. And as a final step, a (6) data validation was performed to assess to assess credibility of the final outputs.

### Defining an area for analysis

Unique *analysis areas* were identified for the contiguous United States (CONUS), Hawaii, Alaska, and the Island Territories (American Samoa, Guam, Northern Mariana Islands, Puerto Rico, and the US Virgin Islands). These polygons were used to determine the processing extent of the model workflow. To determine the analysis area, state shoreline shapefiles (polyline features) were buffered 5 miles (8 km) seaward from the coast. Then, for CONUS and Alaska, a 100-mile (160.9 km) interior buffer was used to create a polygon large enough to cover the entire Floridian Peninsula but exclude most of the United States mainland and the interior of Alaska. For Hawaii and the Island Territories, standard coastal boundaries were used and applied a 0.5-mile (0.8 km) seaward buffer and an interior buffer capable of covering the entire landmass. Each CONUS and Alaska analysis area therefore spans an area 5 miles (0.8 km) seaward and 100 miles (160.9 km) landward from standard state shoreline features. Island territories follow the same logic but extend seaward only 0.5 miles (0.8 km). (Buffer size was not related to the analysis but rather to ensure visually cohesive final products.)

### Preparing a digital elevation model

A mosaic raster Digital Elevation Model (DEM) with 30-meter resolution was created using GeoTIFFs downloaded from The National Map^14^ and clipped to the processing domains described above. The vertical datum for Hawaii, Puerto Rico, the U.S. Virgin Islands, and the Pacific Island territories, are referenced to local mean sea level; however, CONUS and Alaska use NAVD 88 as their reference datum. Therefore, the CONUS and Alaska DEMs were adjusted such that a value of 0 represents local mean sea level. This was accomplished by using NOAA mean sea level elevation data from tide gauge stations (expressed in NAVD 88) downloaded from the United States Army Corps (USACE) tool, the Sea-Level Rise Change Calculator^15^ (now superseded by SLAT) to create a point dataset that was used to generate a Triangular Irregular Network (TIN), where each node represents the mean sea level value of the tide gauge expressed in NAVD 88. The TIN was converted to a raster and subtracted from the original DEM values, to create Local Mean Sea Level (LMSL)-adjusted DEMs where a 0 value represents a close approximation of local mean sea level in the year 2000 (the same baseline used by the NOAA source projections). Equal area projections were used to preserve area and facilitate analysis using the finished products.

### Using Triangulated Irregular Networks (TINs) to create raster files from SLR point projection data

We again used the TIN methodology to incorporate the 2022 NOAA projections (“SLR data”) into our workflow. The SLR data was originally made available in tabular format, where projection values were calculated for tide gauge locations and for a 1-degree grid of interpolated values along all US and outlying territorial coastlines. The SLR data included latitude, longitude, and the median, 17^th^ and 83^rd^ percentile projections of relative sea-level rise for each of the five scenarios (Low, Intermediate-Low, Intermediate, Intermediate-High, and High) and 14 time horizons (i.e., decadal from 2020 to 2150) for a total of 70 combinations for each projection point. All of our outputs use the median percentile projection and omit the 17th and 83^rd^ options. As in the process described above to create the LMSL-adjusted DEM, the point data was used to create TINs for each of the 70 scenario/decade horizon combinations, which were converted into SLR rasters with a grid structure identical to that of the LMSL-adjusted DEMs and then clipped to the respective processing domains.

### Creating gridded SLR projections

The input files described above were combined to determine the areas below sea level for each of the 70 scenario/decade horizon combinations using the following formula:

1 $$\left({{DEM}}_{{LSML}}-\left(\frac{{SLR}\,{Raster}}{100}\right)\right)\cdot 3.28084$$

where ${{DEM}}_{{LSML}}$ is the value of the DEM adjusted by local mean sea level; ${SLR\; Raster}$ is the NOAA projections at tide gauges interpolated using TINs; and unit conversions result in a water depth expressed in feet, given that the primary audience for this effort is U.S. adaptation professionals. From each output, all values less than or equal to 0 were selected, with negative values indicating the change in water depth from local mean sea level in the year 2000.

Here the modeling process bifurcated to create two related modeled outputs for each scenario/decade horizon combination: raster layers indicating the change in water depth due to the rise in mean sea level and polygon layers showing only the extent of mean sea level regardless of water depth.

The spatial extents of the raster outputs were constrained to shoreward pixels. This was done for visual clarity and to exclude artifacts that appear offshore, e.g., raster values indicating the depth of the open Atlantic Ocean as 1 ft (0.31 m). Restricting the raster data to land also reduces the total file size, minimizing data storage requirements, processing, and rendering times and simplifying visualizations. Open water areas were retained for the polygon layers because the data storage requirements for that format are simply not as cumbersome.

### Preserving hydrologic connectivity

**Fig. 4:**
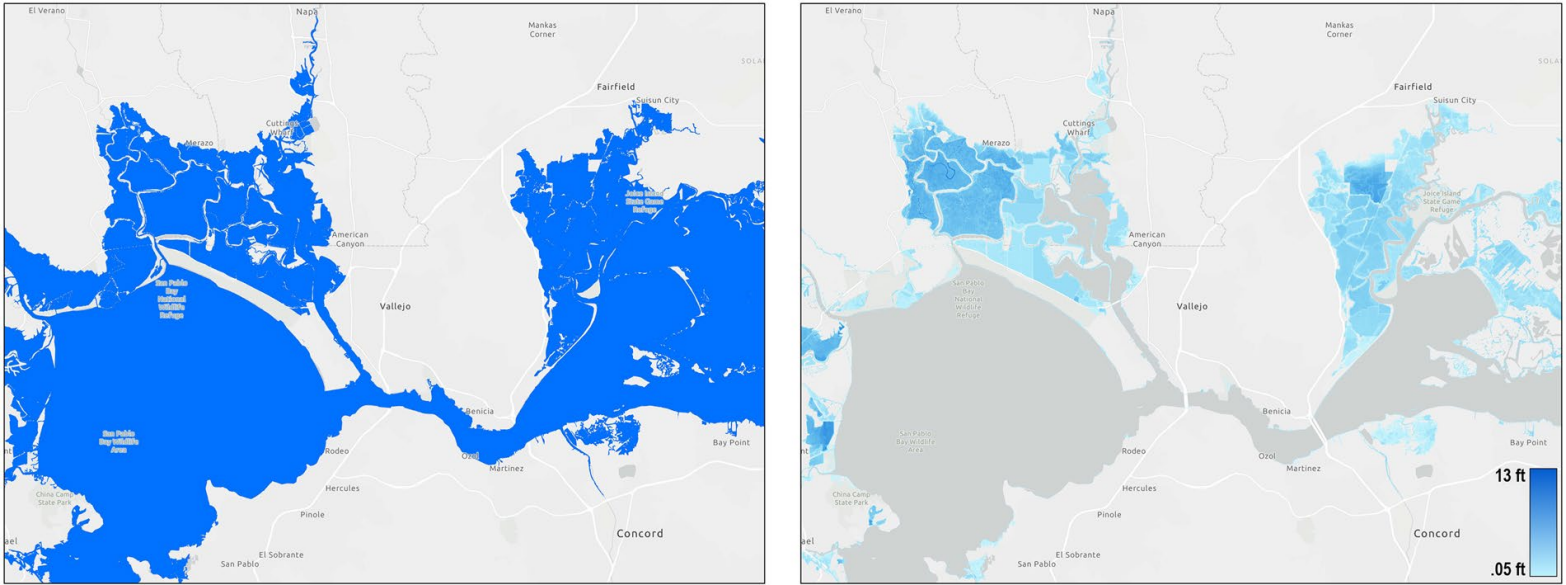
Data outputs from this work include polygon (inundation extent; left) and raster (inundation depth; right) visualizations. Depicted is a location near San Francisco in the year 2100 under the intermediate scenario (1 meter increase in global mean sea level).

The final phase in processing this dataset involved limiting the modeled outputs to include only those areas that are hydrologically connected to the ocean, such as tidal rivers and adjacent floodplain areas. However, connectedness needs to be assessed in a way that accounts for features of the DEM that may artificially create the appearance of disconnectedness, such as bridges, which may be large enough to register on the DEM, or areas that would show up as connected if a finer resolution DEM were used. The result of this process is a set of refined inundation area and depth layers that only include areas that would be directly impacted by sea-level rise (Fig. 4). It also eliminates data anomalies from shallow marine settings that arise from creating the LSML-adjusted DEM without the need to merge it with bathymetry datasets.

The first step involved extracting open water pixels from the National Land Cover Database (NLCD)^16^ raster for each processing domain. All pixels within this area designated as open water were extracted and converted to polygons, capturing both freshwater and marine environments. To eliminate freshwater environments from the data subset, a new polygon was created by erasing the analysis area from the analysis area with a buffer (see step 2) and selecting all NLCD polygons that intersected with this new polygon. Since the analysis area represents terrestrial environments, this new polygon represents marine environments within the analysis area. An additional set of polygons within 0.125 miles (0.2 km) was appended to the initial selection, thus bridging gaps in the NLCD data towards visualizing true hydrological connectivity and producing a Modified NLCD Open Water Polygon to use in the next step.

The second step used the Modified NLCD Open Water Polygons to select a subset of the polygons present in the “modeled outputs” datasets produced in step 4. The selection process included an additional 31-meter buffer to eliminate any remaining single-pixel gaps in hydrologic connectivity (pixels are 30 m; 31 m radius closes this gap). The “cleaned up” sea-level rise projection polygons were merged with the Modified NLCD Open Water Polygons to produce a smooth, visually appealing marine border for the final polygon layers. The final polygon layers were subsequently used to select the hydrologically connected pixels from the “modeled output” raster datasets.

## Data Record

The sea-level rise projections are available in six ESRI File Geodatabases (FGDB) covering all coastal areas of the United States and territories (CONUS, Alaska, Hawaii, Puerto Rico/US Virgin Islands, Guam/Northern Mariana Islands, Samoa): 10.5281/zenodo.15265447^9^.

The sea-level rise projections are also available by EPA region. All databases include the five scenarios of sea-level rise described in the 2022 NOAA report for each decade from the year 2020 through 2150.

## Technical Validation

We compared the NOAA SLRV “Mean Higher High Water + 0 ft” data layer (“NOAA layer”) and the sea-level rise scenario/decade horizon layer (“EPA layer”) with the nearest total water height value at twenty tide gauge locations across the United States (Fig. 1). We expected that there would be substantial overlaps between the datasets, even though the inundation modeling was based on different factors (i.e., sea-level rise versus tide height). The height difference between the NOAA and EPA layers was calculated by subtracting the MHHW height at local mean sea level from the projected sea-level rise height at each tide gauge location. For this analysis the difference ranges between −5.9 and 7.2 cm (−2.3 and 2.8 in), with most differences less than 3 cm (1.1 in) (Table 1).

**Table 1:** Table data indicates percentage of areal overlaps between the specified layers.

TIDE GAUGE:	INTERSECTION TO UNION RATIO, 25 MILES (40.2 KM)	INTERSECTION TO UNION RATIO, 50 MILES (80.5 KM)	INTERSECTION TO UNION RATIO, 100 MILES (160.9 KM)	AVERAGE INTERSECTION TO UNION RATIO	DIFFERENCE IN BASE REFERENCE HEIGHT (MHHW-SLR) (CM)
ANNAPOLIS, MD	*0.975*	*0.947*	*0.913*	*0.945*	*−0.1*
BRIDGEPORT, CT	*0.987*	*0.959*	*0.958*	*0.968*	*−3.2*
CAPE MAY, NJ	*0.932*	*0.886*	*0.869*	*0.896*	*0.8*
CHARLESTON, SC *	*0.938*	*0.876*	*0.837*	*0.884*	*−2.4*
DAUPHIN ISLAND, AL	*0.984*	*0.959*	*0.948*	*0.964*	*−0.5*
DUCK PIER, NC	*0.982*	*0.970*	*0.958*	*0.970*	*−2.7*
FERNANDINA, FL	*0.863*	*0.834*	*0.820*	*0.839*	*0.7*
FRIDAY HARBOR, WA *	*0.982*	*0.965*	*0.965*	*0.971*	*2.8*
GALVESTON, TX *	*0.942*	*0.894*	*0.859*	*0.898*	*−0.3*
LA JOLLA, CA	*0.976*	*0.980*	*0.973*	*0.976*	*1.2*
LIME TREE BAY, USVI	*0.852*	*0.837*	*0.837*	*0.842*	*−0.6*
MOKUOLOE, HI	*0.903*	*0.913*	*0.872*	*0.896*	*−0.4*
PORT ORFORD, OR	*0.960*	*0.951*	*0.950*	*0.954*	*0.1*
PORTLAND, ME	*0.965*	*0.964*	*0.972*	*0.967*	*3.2*
SAN FRANCISCO, CA	*0.949*	*0.823*	*0.742*	*0.838*	*3.9*
SAN JUAN, PR *	*0.763*	*0.772*	*0.778*	*0.771*	*−0.3*
SAN LUIS OBISPO, CA	*0.984*	*0.985*	*0.988*	*0.986*	*−5.9*
ST PETERSBURG, FL	*0.952*	*0.954*	*0.928*	*0.945*	*0.3*
TOKE POINT, WA	*0.941*	*0.921*	*0.795*	*0.886*	*7.2*
VIRGINIA KEY BISCAYNE, FL	*0.951*	*0.819*	*0.819*	*0.863*	*−0.4*
*MIN*	***0.763***	***0.772***	***0.742***	***0.771***	***−5.9***
*MAX*	***0.987***	***0.985***	***0.988***	***0.986***	***7.2***
*AVG*	***0.937***	***0.909***	***0.888***	***0.911***	***0.17***

“Intersection” refers to the shared overlapping area between MHHW (NOAA) and SLR (EPA) data, whereas “Union” refers to their combined area when joined together. A value of 1 would represent a completely coterminal area of coverage. Starred* entries correspond to the tide gauges used in Fig. 8.

We also calculated and compared the total area of inundation for the NOAA and EPA layers within concentric buffers at 25, 50 and 100 miles (40.2 km, 80.5 km, 160.9 km) from tide gauges (Fig. 1). We calculated the ratio of the area of intersection between the two layers (i.e., both layers indicate that a pixel is inundated) to the combined total area of inundation indicated by both layers (i.e. NOAA inundation area plus EPA inundation area). Visual examples of this analysis are provided in Fig. 6, and more detailed statistics are provided in Table 1 and Fig. 7. The results of this comparison show that in the majority of cases the ratio values are greater than 90%, with nearly half of the values overlapping at 95% or better. This indicated an overall strong agreement between the two data sources. We note that dataset agreement decreases slightly as the area around the tide gauge increases; the average overlap across all twenty locations is 94% within 25 miles of the tide gauge and decreases to 89% when considering a 100-mile radius.

**Fig. 5:**
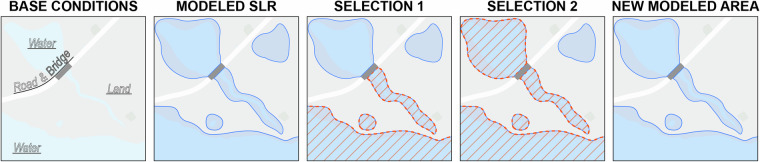
Frame 1 represents current land and water areas. Frame 2 shows all modeled SLR inundation areas after step 2.4 and before adjusting for hydrologic connectivity. Frame 3 shows the first selection, which selects modeled SLR areas that intersect with the ocean plus anything with 0.125 miles (~200 m) of that selection to account for bridges or other features that may artificially truncate hydrologic connectivity. Frame 4 expands the selection to any SLR areas within 31 m of the first selection to account for hydrologic connectivity that may be lost with 30 m DEM resolution that wouldn’t be lost if the DEM data were finer-scaled (e.g., 1 m).The NLCD is not available for the Pacific Island Territories, the Hawaiian island of Ka’ula and the Puerto Rican islands of Isla de Mona and Isla de Desecheo Marine Reserve. Consequently, hydrologic connectivity processing was not completed for these areas.

**Fig. 6:**
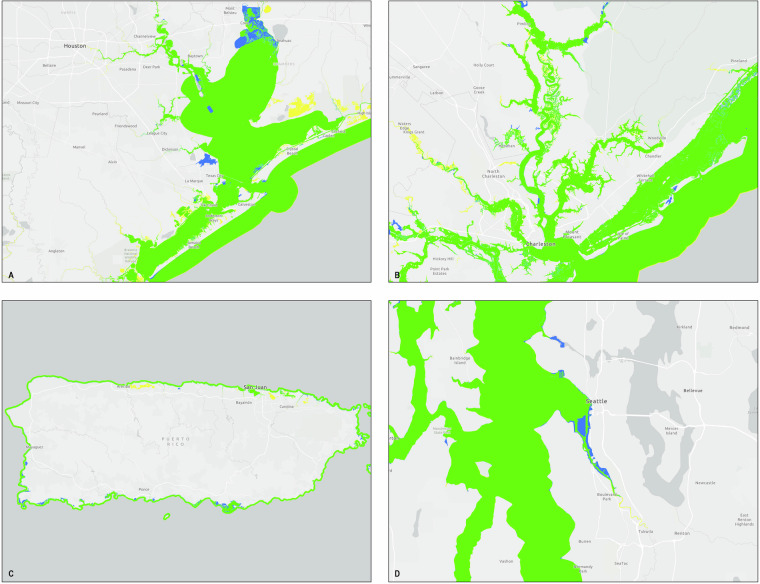
To validate data, a comparison was made between NOAA’s “Mean Higher High Water + 0 ft” data layer and the sea-level rise scenario/decade horizon layer with the nearest total water height value. Overlap between the datasets is indicated by green shading, while blue shading indicates areas captured only in the datasets generated for this paper, and yellow indicates areas captured only in the NOAA Sea-Level Rise Viewer datasets. Shown are (**a**) Galveston, TX (Low Scenario in 2020), (**b**) Charleston, SC (Intermediate-Low Scenario in 2120), (**c**) Puerto Rico (High Scenario in 2040), and (**d**) Seattle, WA (Intermediate Scenario in 2110).

**Fig. 7:**
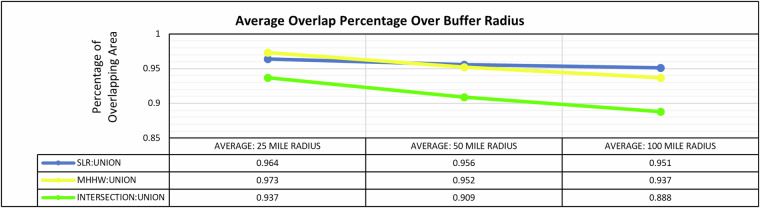
Similarity in inundation extent by each of the datasets. Union refers to the union of NOAA’s “Mean Higher High Water + 0 ft” data layer (MHHW layer) and the sea-level rise scenario/decade horizon layer (SLR layer) within the specified distance from a tide gauge (25, 50 and 100 miles / 40.2 km, 80.5 km, 160.9 km. Intersection refers to the intersection of the same datasets. SLR, MHHW, and intersection polygons were compared to the union of the datasets and indicate an overall high overlap but with a slight decrease as distance from the tide gauge increases.

The maps in Fig. 6 also highlight some of the limitations of inundation exposure analysis and by extension the visualizations produced for this manuscript. For example, Panel 6 A illustrates the area around Galveston, TX, with green indicating where both the NOAA MHHW and EPA SLR data project open water; however, a large area in the north of the panel is shown in blue, indicating inclusion in the EPA SLR projections but not the NOAA MHHW extent, while in the east the opposite is true (shown in yellow). In satellite imagery, the large areas shown in yellow and blue are coastal wetlands, which typically have elevations very close to mean sea level. Additionally, emergent vegetation can produce anomalous values in elevation datasets. Both of these factors can produce more uncertainty in visualizing the extent of sea-level-rise inundation. From a decision-making perspective, these differences may be less critical, since they occur in locations where infrastructure is unlikely to be sited.

Panel 6C illustrates the island of Puerto Rico and highlights another challenge in making direct comparisons between the inundation caused by SLR versus that caused by MHHW. The San Juan tide gauge in the northeast quadrant of the island has a MHHW value of 0.81 ft (0.25 m), but the tide gauge at Magueyes Island in southwest Puerto Rico has a MHHW value of 0.34 ft (0.10 m). Other nearby tide gauge stations in the US Virgin Islands (Charlotte Amalie and Lime Tree Bay, also on the south sides of their respective islands) have MHHW values very similar to that of Magueyes Island (0.34–0.41 ft [0.10–0.12 m]). In this example the High scenario in 2040 was used to compare inundation extents. In San Juan the median sea-level-rise projection is 0.82 ft (0.25 m), which is nearly identical to the MHHW value at that location; sea-level-rise projections for the other stations are very similar to that of San Juan and range from 0.79–0.82 ft (0.24–0.25 m). In other words, the comparison will produce better results (i.e., better overlap) around San Juan than along the southern coast of the island, because the SLR values are similar across the island while MHHW values are lower in the south. This is reflected in Fig. 6 by more SLR-dominated inundation coverage (blue areas) appearing along the southern and western coasts. The combination of exposure to different tidal dynamics across relatively short distances and the relatively sparse distribution of tide gauges can explain why this location had the lowest overlap in the analysis (0.771, Table 1). Similarly, the overlap around the Friday Harbor tide gauge station near Seattle, WA is very high (0.965–0.982) and captures inundation in the Puget Sound region. By contrast, the tide gauge at Toke Point, WA is located on the Pacific coast and displays high overlap (0.921–0.941, Table 1) until the buffer is expanded to 100 miles, at which point it drops significantly (0.795, Table 1); at 100 miles, the buffer includes part of the Puget Sound, supporting the interpretation that differing tide dynamics play a role in shaping inundation patterns.

These results are not wholly unexpected when one considers that changes to water height caused by SLR tend to be less spatially variable than tides, which are heavily influenced by coastal geomorphology. The decrease in overlap with increasing distance from the central tide gauge can also be driven by factors such as differences in baseline raster resolutions, polygon simplification after conversion from raster formats, and differences in models of measuring hydrologic connectivity, all of which creates some variability in the spatial extent depicted in the datasets. Although imperfect, these comparisons offer a useful way to test the reasonableness of our visualizations against other similar high-quality datasets.

**Fig. 8:**
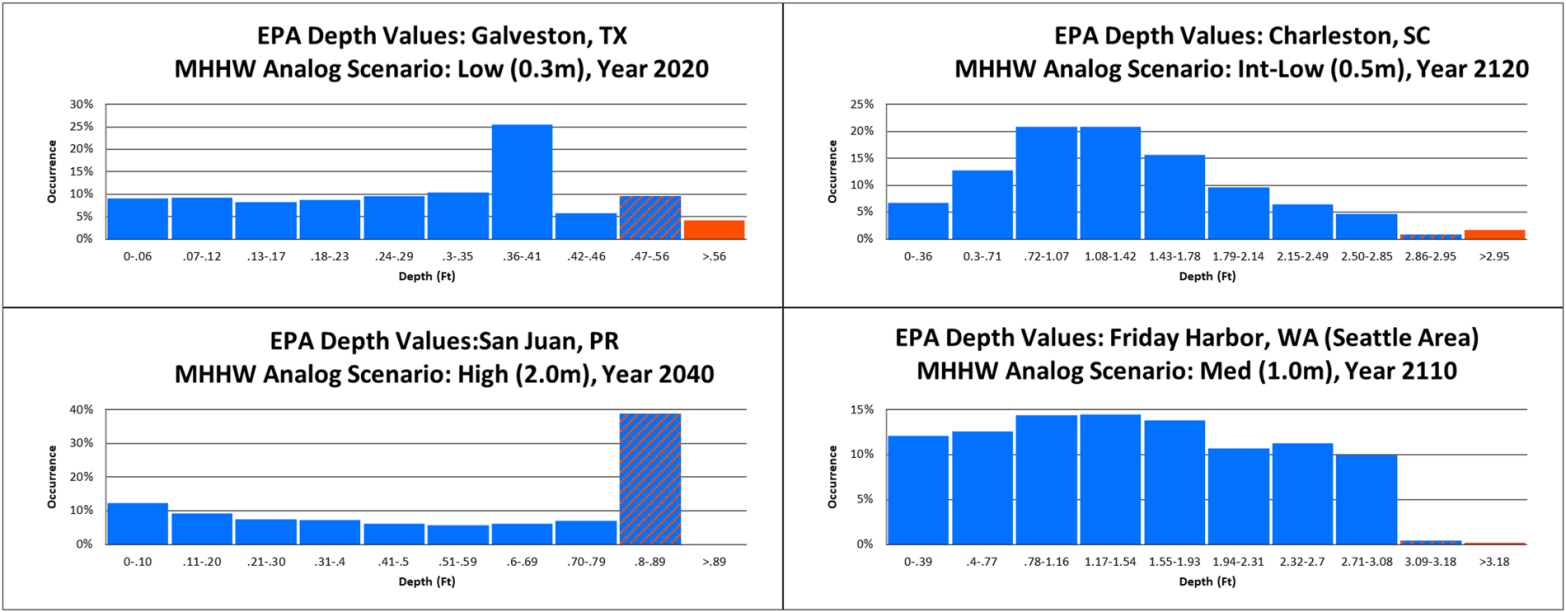
Bar charts indicating the distribution of depth values within five miles (8.05 km) of a tide gauge. Blue bars (first eight bars in each panel) represent depth values up to and including the sea-level rise projection for the scenario/decade combination that most closely matches the MHHW analog used for that location (0.46 ft (0.14 m) in Galveston, TX; 2.85 ft (0.87 m) in Charleston, SC; 0.79 ft (0.24 m) in San Juan, PR; and 3.08 ft (0.94 m) in Friday Harbor, WA). (See Fig. 6 and Table 1 for more information.) The ninth semifinal bar (hatched orange/blue) represents values within 0.1 ft (3 cm) of the threshold in bar eight. The final bar (orange) represents values which exceed the expected range + 0.1 ft (3 cm) threshold. Results indicate that the depths represented in the dataset are within expected values.

A final validation step tested whether pixel values in the raster datasets fall within the expected range of water depth for the scenario/decade combination that most closely matches the MHHW analog used for that location. Four sites from disparate locations were chosen from the list of tide gauges in Table 1 and water depth values within a five-mile radius were tabulated and arranged into a histogram representing counts of values within the specified range (Fig. 8). Results indicate that the majority of pixels have values within the expected range (blue bars in Fig. 8; location-specific thresholds noted in figure caption) and that values that exceed this range fall mostly within 0.1 ft (3 cm) of the maximum range value (hashed bar in Fig. 8). The small number of pixels with values beyond this range (orange bar in Fig. 8) are associated with areas below sea level in the DEM, including natural topography and human-created features such as open pit mines. The highest number of out-of-range values was identified in Galveston, TX and totaled 4.1% of pixels, indicating that most values fall within a reasonable tolerance of the expected range. The target threshold value is the approximate cap on expected values, but it is not an absolute maximum, as variations in the production of the SLR scenario rasters and its underlying data, small discrepancies in target reference values, and certain landforms can produce data that slightly exceeds this range. Therefore, a distribution of values that generally falls below the target reference value with a smaller portion of exceedance is indicative of the overall accuracy of the pairing process.

### Use and limitations

The data products described above possess several important limitations. First, since our work relies on pre-existing sea-level rise projections, our outputs necessarily include the assumptions and limitations of that work. The 2022 NOAA projections take into account factors that affect mean sea level at large scales, such as sterodynamic sea level change, gravitational, rotational, and deformational changes due to ice loss, vertical land motion, and more, but do not take into account factors that occur predominantly at smaller scales, such as erosion, sedimentation, or many human-made interventions. Additionally, the range of projections around the median value is larger in later decades. As such, the projections are less likely to represent the extent of mean-sea-level inundation the further in the future they occur. For a full list of the factors included in the creation of the source dataset as well as uncertainty, updates to previous models, and extended usage notes, please refer to the 2022 NOAA technical report^2^.

Second, the model used to create this dataset accounts for hydrological connection by selecting contiguous areas below the given threshold for each scenario/decade combination, i.e., our approach incorporates the “bathtub model” of flood dynamics. Unlike some bathtub models, our approach ensures that the final output represents bodies of water that are currently connected to the ocean by successively adding valid areas that are less than two pixels (31 m) from the initial selection and excluding negative valued pixels which do not connect to the ocean (Fig. 5). This accounts for errors in the DEM which may have inadvertently collected bridges or other apparent obstacles and assigned them inaccurately high pixel values (which would create an artificial disconnection in this analysis), but it does not substitute for a more rigorous model of local hydrology that can account for shoreline erosion, water flow dynamics, and surface materials. Additionally, we make no assumptions about future flood protection projects nor do we manually include existing structures not represented in the original DEM, such as large dams. Users interested in examining the role that human intervention might play, or have already played, in planning for exposure to sea-level rise might use this tool to understand the present-day baseline of exposure that an area may face but would need to do additional research to determine if and how a response might be expressed in the built environment.

The limitations listed above describe a tool which is best used to quickly examine a site or region to understand the range of scenario/decades which may impact that site. Anyone who lacks the scientific expertise to navigate more complex modeling processes may be better served by the orientation of the data towards the scenario/decade format. This audience includes community groups, non-academic researchers, or professionals involved in adaptation planning at a large scale or early in project development. Users such as state and municipal officials who might be interested in examining large regions or multiple disparate sites across regions can use this data to screen large areas for a range of viable sites, compare the relative exposure to sea-level rise across those sites, or look to determine when and how a range of sites might be inundated beyond a critical threshold. Users should carefully consider whether their use case necessitates additional analysis or modeling and acknowledge the inherent uncertainty of these projections in their conclusions.

## Usage Notes

The sea-level rise projections may be accessed interactively at https://seatool.epa.gov or downloaded at https://zenodo.org/records/15265448. The data format for all projections is ESRI File Geodatabase (FGDB).

## Data Availability

The data created through this research are available through a Zenodo repository: 10.5281/zenodo.15265448. “2022_SLR_Data_CONUS.gdb.zip” contains all SLR data for the continental United States. Data is also broken up into 12 additional ESRI File Geodatabases which contain the data separated into coastal EPA regions (Regions 1, 2, 3, 4, 6, 9, and 10) as well as Hawaii (Region 9) and the US Territories of Puerto Rico / US Virgin Islands (Region 2 Caribbean), Guam / Northern Mariana Islands (Region 9), and Samoa (Region 9). All geodatabases include vector-based polygon shapefiles showing covered area (File Geodatabase Feature Class) and raster files (File Geodatabase Raster) for water depth. File nomenclature is in “REGION_Location_Type_Scenario_Year” format, where “R9_Guam_NMI_Area_D2022_S030_Y2120” describes a location in Region 9, Guam and Northern Mariana Islands, in area (vector-based polygon) format, from the 2022 projection source data, in a 0.3 m GMSL scenario, for year 2120. Each geodatabase contains files for each of the 5 SLR scenarios in decades 2000–2150.

## Code availability

The sea-level rise projections were generated using ArcGIS Model Builder. That ArcGIS Toolbox and a figure depicting workflow are available at https://zenodo.org/records/15212258.
